# Advanced Localization Technique for Non-Palpable Breast Cancer: Radiofrequency alone VS Combined Technique with Ultrasound

**DOI:** 10.3390/jcm12155076

**Published:** 2023-08-02

**Authors:** Simona Parisi, Claudio Gambardella, Giovanni Conzo, Roberto Ruggiero, Salvatore Tolone, Francesco Saverio Lucido, Francesco Iovino, Francesca Fisone, Luigi Brusciano, Domenico Parmeggiani, Ludovico Docimo

**Affiliations:** 1 Division of General, Oncological, Mini-Invasive and Obesity Surgery, University of Campania “Luigi Vanvitelli”, 80131 Naples, Italy; claudio.gambardella2@unicampania.it (C.G.); salvatore.tolone@unicampania.it (S.T.); francescosaverio.lucido@unicampania.it (F.S.L.); francesca.fisone@unicampania.it (F.F.); luigi.brusciano@unicampania.it (L.B.); domenico.parmeggiani@unicampania.it (D.P.); 2 Department of Advanced Medical and Surgical Sciences, School of Medicine, University of Campania “Luigi Vanvitelli”, 80131 Naples, Italy; ludovico.docimo@unicampania.it; 3 Department of Traslational Medical Sciences, University of Campania “Luigi Vanvitelli”, 80131 Naples, Italy; giovanni.conzo@unicampania.it (G.C.); francesco.iovino@unicampania.it (F.I.)

**Keywords:** breast localization, radiofrequency localization, LOCalizer, ultrasound breast localization, early breast cancer

## Abstract

Breast conservative surgery is the primary therapeutic choice for non-aggressive early breast cancers, and a minimally-invasive approach is strongly recommended. The breast localization represents a modern challenge for surgery. Wire-guided localization is still the gold standard technique, but new wireless systems have been proposed, such as radiofrequency identification with LOCalizer^TM^ (Hologic, Santa Carla, CA, USA), which reports encouraging results. The current study aimed to evaluate the accuracy and efficacy of the combined use of LOCalizer^TM^ and ultrasound compared with the results obtained using LOCalizer^TM^ alone for the detection of non-palpable breast cancer. Ninety-six patients who were candidates for breast localization were enrolled. Group A received a combined localization with LOCalizer^TM^ and US, while group B underwent only LOCalizer^TM^ identification. Oncological radicality was reached in 100% of the patients in Group A and in 89.2% of the patients in Group B, with *p* = 0.006. The mean specimens’ volume was 13.2 ± 0.6 cm^3^ for Group A and 16.1 ± 1.4 cm^3^ for Group B, while mean specimen weights were 21.8 ± 2.2 and 24.4 ± 1.8 g, respectively (*p* = 0.003 and *p* = 0.004, respectively). LOCalizer^TM^ with ultrasound, in the current series, has resulted in the preferred option for the localization of non-palpable breast cancer, allowing limited resection (in weight and volume), guaranteeing excellent oncological outcomes, and great satisfaction for patients and physicians.

## 1. Introduction

Breast cancer (BC) is the most common worldwide malignancy affecting women and the leading cause of cancer deaths [1]. The introduction of mammographic screening has significantly decreased morbidity and mortality due to the early identification of BC [2]. The spread of innovative technologies such as Tomosynthesis, Elastosonography, the Contrast Enhancement Spectral Mammography (CESM), and Artificial Intelligence Computed-Aided Detection contributed to a further improvement of early BC detection and to revolutionary changes in diagnosis, oncological treatment, and surgical management. Moreover, the key role of breast surgery as primary therapy is increased, and the removal of the early BC is recommended by international guidelines [3]. Contrary to the past, when radical demolition surgery was largely appreciated and advocated, nowadays greater emphasis is placed on achieving acceptable cosmesis; breast surgeons aim to perform mini-invasive approaches to reduce the impact of local excision on cosmesis. Moreover, the actual state-of-art for oncological radicality validated in the Saint Gallen Consensus in 2015 aimed to guarantee the “absence of ink on tumor” for invasive BC while it is necessary only a 2 mm distance between the in-situ BC and the specimen’s margin [4]. Therefore, healthy tissue demolition should be demonized and mini-invasive surgery for early BC is auspicated. It is estimated that 30% of BC are non-palpable lesions at the diagnosis and the necessity of modern breast localizing technique is a key element in breast surgery. Wire-guided localization (WGL) represents still the gold standard technique. 

It involves inserting a wire into the breast parenchyma to target the non-palpable lesion under mammographic or ultrasound guidance. The main advantages are the low cost, the familiarity with the technique, and the availability. Several limitations have been raised, such as patient discomfort, breakage, migration, and infection. Moreover, it is usually necessary to perform the wire placement on the same date of the surgery [5]. New wireless localizing systems have been proposed, such as radiofrequency identification with LOCalizer^TM^ with encouraging results [6]. 

There are other localization modalities reported, many of which were used in the experimental setting. LOCalizer^TM^ (Hologic, Santa Carla, CA, USA) system is characterized by an interesting exclusive feature: the detection of distance in millimeters from the tagged lesion to the surgical probe. It is possible because the marker named Tag is a microchip that emits radiofrequency waves when stimulated. Although the main endpoint for the surgeon is the oncological radicality and the identification of the NPBC radiological margins, not the marker position. The radiologic margins may be defined as the interface between the hypoechogenicity of the lesion and the surrounding parenchyma, which appears more echogenic. Moreover, the Tag placement can be realized by releasing the marker perfectly into the lesion, but it is often collocated near or with only a part of the lump. Therefore, we proposed in a previous pilot study a combined technique with LOCalizer^TM^ and Ultrasound (US) to increase BC margins’ detection and cosmetic results [7,8]. 

The current study aimed to evaluate the accuracy and efficacy of the combined use of LOCalizer^TM^ and US compared with the results obtained using LOCalizer^TM^ alone for the detection of non-palpable BC (NPBC). 

## 2. Patients and Methods

### 2.1. Study Design

This study is reported according to the STROBE statement for cohort studies [9]. A retrospective study was conducted to compare the feasibility of the combined use of LOCalizer^TM^ and US with LOCalizer^TM^ alone for the detection of NPBC. It was conducted according to the ethical principles stated in the Declaration of Helsinki. Written informed consent was obtained from all subjects.

### 2.2. Study Setting and Study Population

From June 2020 to December 2022, all the patients affected by breast cancer referred to the Division of General Surgery of a Teaching Hospital were considered for enrollment in the study. Inclusion criteria were age ≥18 years and the presence of an early non-palpable BC (T1 according to TNM classification [10]) confirmed by a core biopsy. Patients affected by in situ BC and multiple microcalcifications were excluded because of the high risk of multicentric extension. 

Preoperative evaluation included a recent (not more than six months old) breast clinic exam, bilateral mammography, and a breast US. An accurate explanation of the necessity of breast localization and all possible options was performed. The patients accepting radiofrequency localization with LOCalizer^TM^ and US signed the informed consensus and were addressed to Tag placement.

All the surgeries were performed by experienced breast surgeons (over 500 procedures).

Clinical data were collected in a prospective electronic database. Patients who received the combined localization with LOCalizer^TM^ and US were considered in Group A, and patients receiving the breast localization with LOCalizer^TM^ alone were considered in Group B.

### 2.3. LOCalizer^TM^

**Figure 1:**
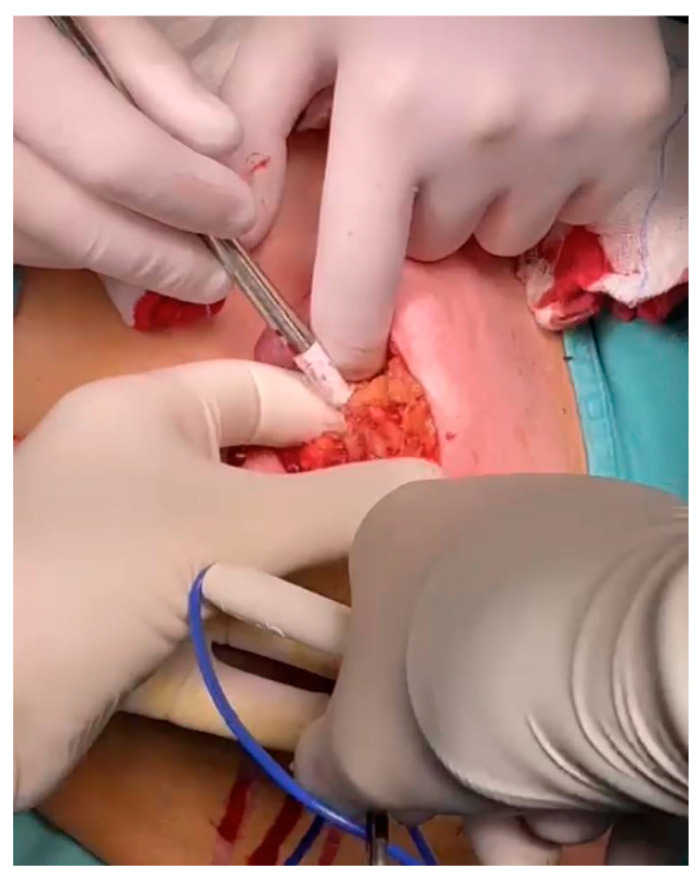
Breast localization withLOCalizer^TM^.

LOCalizer^TM^ is a composite device composed of the following elements: a Tag, an RFID chip; an Applicator, a 12-Gauge-needle; a Reader, a console with a display; Pencil, a very handily surgical probe able to activate the Tag into the lesion. Each Tag measures about 1 cm and is associated with a unique identification number, which appears very useful in the case of two or more lesions to excise. The Tag transmits its digital data only when triggered, releasing an acoustic signal and indicating its distance in mm on the Reader display. The pitch and volume of the sound increase when the Pencil approaches the Tag (Figure 1).

### 2.4. Surgical Technique 

The placement of the Tag was performed 15 days before the planned surgery. Tags were placed by introducing the percutaneous 12-gauge Applicator under US, with local anesthesia. The Tag placement aimed to release the marker perfectly into the lesion, but it can be collocated near or only partially into the lump. On the surgery day, the patients were placed in a supine position on the surgical table, with both arms abducted. A general anesthesia was performed. 

Group A. The NPBC localization was realized with LOCalizer^TM^ combined with US. SuperSonic^®^ MACHTM 30 (Hologic, Santa Carla, CA, USA) is the Ultrasound machine engaged for the study, with a 5–18 MHz conventional linear transducer with a footprint face of 51 mm. The operator (a surgeon with certified ultrasonographic intraoperative experience in more than 300 procedures) used a B-Mode scan and the option to measure the distance between two points. The US was performed newly, right after the placement, to evaluate and record the following parameters, globally named “TM (Tag-Margin)” distances. Cranial, caudal, medial, lateral, upper, and lower NPBC margins were considered, and their distance to the Tag was calculated. The reference points on the Tag and in the margins were always the midpoints for each side. When the Tag midpoint was out of the lesion, it was recorded before the minus sign. On the surgery day, US scans were performed to evaluate any possible migrations, and the TM parameters were newly controlled. Furthermore, the Reader and US were employed to search the incision site. Once the surgeon has reached the breast parenchyma, the Pencil is used to identify the Tag, following the sound intensity and the distance reported on the Reader Display. This latter is defined as “TP (Tag-Pencil)”. Coming from cranial, caudal, medial, lateral, upper, and lower directions, the surgeons compared the recorded US-measures “TM”, with the millimeters reported on the display “TP”. In this way, they could know the distance between the Pencil and the margin, named “PM (Pencil-Margin)”, through the subtraction: “TP − TM = PM”. After the excision, they verified the absence of a signal in the residual parenchyma and the presence of the Tag in the specimen. New coordinates TM were measured to obtain the first immediate confirmation of oncological radicality. Furthermore, the specimen was sent to pathologists. The time consumed was calculated from the surgical incision to the sending of the specimen, including the last post-excisional US evaluation. 

Group B. The NPBC localization was realized with LOCalizer^TM^. US scans were performed during the placement and right after the Tag release. On the surgery day, a US exam was performed only to verify any possible migrations. A reader was employed to search the incision site. Once the surgeon has reached the breast parenchyma, the Pencil is used to identify the Tag, following the sound intensity and the distance reported on the Reader Display. This latter is defined as “TP (Tag-Pencil)”. After the excision, the absence of signal in the residual parenchyma and the presence of the Tag in the specimen were verified. Furthermore, the specimen was sent to pathologists. 

### 2.5. Outcome Measures

Oncological radicality was valued after the surgery with the definitive pathological examination of the specimens sent to the Pathological Institute of the Hospital. According to Saint Gallen’s recommendation, when the cancer margin was not inked at microscopical observation, the surgical radicality was obtained and reported as R0 surgery on the pathological report. 

Only for group A (combined LOCalizer^TM^ and US localization), an intraoperative evaluation was performed on the specimens just after the excision, comparing “TM distances” with TP values. If TP was equal or major, then TM, the asportation was considered satisfactory. If not, a re-excision was realized. Equally, the specimens were sent to the Pathological Institute. 

The Migration phenomenon consists of a shift of the Tag from the site of the placement. For both groups, the preoperative US scan reporting the NPBC and the Tag was compared with the post-placement image. A shift greater than 5 mm was considered suggestive of migration. 

The operative time was calculated in minutes from the skin cut to the NPBC excision.

The specimen volume was estimated by measuring the three coordinates of the NPBC at microscopical observation: width, length, and height in cm^3^, while the weight was obtained by the pathological report and measured on a Kern FOB-NLO balance (Kern, Balingen, Germany). For both groups A and B, specimens’ weight and volumes (such as parameters correlated to the surgical mini-invasive approach) were evaluated by pathologists, and the values were expressed in milligrams (mg) and cubic centimeters (cm^3^), respectively.

Clinicians’ and patients’ satisfaction was evaluated with a questionnaire 30 days after the surgery. The test investigated the opinion about the compliance to the Tag placement, the anxiety before the surgery, the possible complications, and the cosmetic results according to a Likert scale with a 0–10 point range. Furthermore, clinicians and patients were asked about their overall satisfaction [11].

### 2.6. Study Outcomes 

The primary outcome was the evaluation of oncological radicality at the definitive pathological examination in patients who underwent combined LOCalizer^TM^ and US localization and LOCalizer^TM^ alone for NPBC. Secondary outcomes were the evaluation of the migration rate, the operative time, the volume, and the weight of the excised NPBC. Furthermore, clinicians’ and patients’ satisfaction were investigated. 

### 2.7. Statistical Analysis 

Statistical analysis was performed via Excel 2011^®^ (Microsoft, Redmond, WA, USA) and the Graph-Pad Prism^®^ 9 program (San Diego, CA, USA). Categorical data were reported as raw numbers with percentages in parentheses. Continuous data were reported as means ± standard deviation or as medians with the range in parenthesis, according to the distribution. The differences between results were analyzed by the unpaired t-test if they were summarized as means, the Mann-Whitney U test if they were summarized as medians, or the Fisher’s exact test if they were reported as percentages. A *p* value of less than 0.05 was considered statistically significant.

## 3. Results 

### 3.1. Study Population 

Between June 2020 and December 2022, 106 women with NPBC were referred to the Unit of General Surgery of “Campania University Vanvitelli” (Naples, Italy). Four people did not meet the inclusion criteria, while six refused radiofrequency localization. Ninety-six patients, all women, met the eligibility criteria and were enrolled. Afterward, we set up the combined technique. Sixty-eight women received breast localization with LOCalizer^TM^ and US and were included in Group A. Twenty-eight patients received exclusive LOCalizer^TM^ identification. Baseline demographic and clinical characteristics are reported in Table 1. 

The Tag placement was correctly performed into the lesion in 39.7% of the patients (27/68) in Group A and 39.2% (11/228) in Group B. The Tag was collocated near but out of the lesion in 32.4% of the patients in Group A (22/68), and 17.9% (5/28) in Group B. The marker was placed in part in the NPBC at 27.9% (19/68) in Group A and at 42.9% (12/28) in Group B. In all cases, the difference was not statistically different (Table 2).

### 3.2. Outcomes

The primary aim of this clinical study was to evaluate oncologic radicality after a definitive pathological examination. We correlated the pathological reports to each patient and each procedure, verifying if the Saint Gallen indication (“no ink on the tumor”) was respected and obtaining the surgical radicality. It was reached for 100% of the patients in Group A and for 89.2% of the patients in Group B, with *p* = 0.006. 

The migration rate was a further secondary endpoint. It was established during the preoperative US, with the check of the “TM distances”, for Group A. Only for a patient a shift >6 mm in medial-lower direction was observed. For Group B, on the surgery day, a US exam was performed just to verify any possible migrations as a shift >5mm. No migration was observed.

The operative time was calculated from the skin cut to the NPBC excision. Despite the double technique, for group A, it amounted to 15.7 min compared with 14.5 min for group B. These values show a substantial similarity (*p* = 0.279) and that the combination with US does not delay the surgery times. 

The mean specimen volume was 13.2 ± 0.6 cm^3^ for Group A and 16.1 ± 1.4 cm^3^ for Group B. while the mean specimen weights were 21.8 ± 2.2 and 24.4 ± 1.8 g, respectively (*p* = 0.003 and *p* = 0.004) (Table 3). These data demonstrate that the combined localization with LOCalizer^TM^ and the US is minimally invasive. Better-tailored excisions and cosmetic results were performed. 

Therefore, patients and clinicians’ satisfaction was tested through a questionnaire 30 days after the surgery, when cosmetic results were stable and histological reports were available. According to the patients’ opinions, the combined technique was less invasive and associated with better cosmetic results (9.2 vs. 8.1, respectively, for groups A and B, *p* = 0.003). Furthermore, the surgeons appreciated the LOCalizer^TM^ and US, considering them safer and more precise than the standard use of the LOCalizer^TM^ alone. 

For group A, an intraoperative evaluation of surgical radicality using the LOCalizer^TM^ system and US was performed and compared with a definitive pathological examination. The US scans on the specimens were realized to obtain the new measurement of the “TM distances”. If they were equal or major, then the previous parameter, the asportation was considered sufficient, and no re-excision was performed. In 3/68, the TM distances appeared inadequate, and a little re-excision was performed right away. The definitive histological exams confirmed for each of the 3 cases the presence of ink on the margin and the necessity of the reoperations (re-operation rate for Group B = 10.7%). These latter had already been performed, and the oncological radicality was insured. 

Overall survival is 100%, and no cases of recurrence have been recorded after a mean follow-up of 18 months. 

## 4. Discussion

The breast localization for NPBC can be extremely challenging, especially in breast conservative surgery. Currently, the gold standard technique for breast localization is WGL, but many issues have been raised, such as patient discomfort and the risk of breakage, migration, and infection [12]. Therefore, the technique might be considered outdated, although it is probably cheaper than others. Radioactive seed localization (RSL) has been proposed to avoid the WGL’s limits and, above all, the patient’s anxiety. The seed is approximately 5 mm in size, is implantable into the breast parenchyma, and is generally well tolerated by the patients. However, the surgical procedure should be performed as soon as possible because the radioactive tracer associated with the seed lasts less than 5 days. Noteworthy, the radioactivity also represents a risk for the patient and the operators. Moreover, the technique is pollutant-intensive, and disposal management might be difficult [13]. Further innovative wireless techniques have been proposed and performed; most of them are characterized by the capability to indicate with acoustic signals the marked area. 

LOCalizer^TM^ differs from the other proposed methods by presenting the exclusive feature of showing the exact distance in mm between the surgical probe and the Tag [14]. It is one of the most recent wireless localizing systems and was first tested in 2019 by DiNome M.L. et al. on 50 women, resulting in satisfying results for the absence of migration, the successful removal of all Tags, and the high acceptance among patients and clinicians [11]. Two trials compared the new wireless radiofrequency system to the actual gold standard. Mc Gugin et al. verified a re-excision rate of 19.1% after the LOCalizer^TM^ localization compared with the 16.8% of the WGL group in a large study on 503 procedures [15]. In a study on 83 patients, Lee et al. compared LOCalizer^TM^ with WGL, concluding that the operative times were similar (79 min for LOCalizer^TM^ and 78 min for WGL), while re-excision rates were better using the wireless method (6.1% vs. 10.0%, *p* = 0.82). Moreover, positive margins were reported for 3% of the patients treated with LOCalizer^TM^ and 8% of WGL (*p* = 0.67) [16]. 

LOCalizer^TM^, compared with the other wireless instruments, appears advantageous for its ability to offer the millimetric distance from the target, while most of the others are based only on the emission of acoustic signals. However, LOCalizer^TM^, similar to all the other techniques, cannot detect the distance from the lesion margins, which represents the ideal information for the surgeons, according to the Saint Gallen International Expert Consensus in 2015 [4]. Moreover, the more precious data for surgical radicality is the identification of the cancer margins and not the Tag position. The markers should ideally be released into the lesions, but they are often placed near them or at one of the edges. The simple US examination after the Tag placement can verify the position of the marker. The combined technique LOCalizer^TM^ and US aim to improve margin detection for NPBC to obtain safe oncological treatments and mini-invasive resections. It is based on the possibility of calculating the unknown distance between the margin and the surgical instruments, thanks to the available data: the distance from the Tag offered by the Reader and the distance between Tag and margin offered by the US exam. Moreover, it is possible that the Tag is placed into the lesion but near one of the margins than others, or that the Tag is next to the lesion but outside of it. US detection can contribute to improving the surgeons’ orientation during NPBC excisions. Despite LOCalizerTM being characterized by the abovementioned exclusive and promising features, it could not replace WGL as the gold standard localizing technique. Probably, the relevant costs of the radiofrequency system have an important impact.

Starting from a previous experience with a limited cohort, the current study aimed to evaluate the feasibility and efficacy of the combination of LOCalizer^TM^ with US [7]. To the best of our knowledge, this is the first study comparing the results of the combined localization of LOCalizer^TM^ with the US with LOCalizer^TM^ alone. The primary aim was the oncologic radicality after the definitive pathological examination and it was verified in 100% of the patients undergone to the combined technique vs. 89.2% (25/28) in Group B (*p* = 0.006). These results suggest that the combined technique offers an important advantage for oncological radicality. The operative time was similar (15.7 vs. 14.5 in Group A and B, respectively; *p* = 0.279), confirming that the combined technique is not time-consuming in experienced hands. In Group A, the duration of the post-excision US appears to be compensated by a quicker asportation of the NPBC, probably due to a more precise identification of the lesion’s extension. Moreover, the guided combined localization allowed to perform targeted resections with reduced specimens’ volume (13.2± 0.6 cm^3^ vs. 16.1 ± 1.4 cm^3^ in Group A and B, respectively; *p* = 0.003) and reduced specimens’ weight (21.8 ± 2.2 g vs. 24.4 ± 1.8 g in Group A and B, respectively; *p* = 0.004). These aspects appear of utmost importance since the modern trends in breast cancer are addressed to increasingly minimally invasive resection, paying particular attention also to the cosmetic result and the patient ‘satisfaction, that in our series resulted significantly improved in Group A (Table 3). Furthermore, the clinicians’ satisfaction was significantly higher in Group A, probably for the better awareness of the Tags and margins’ location.

Moreover, thanks to the US check on the specimens in Group A, it was possible to predict the absence of surgical radicality in 3/68 cases (4.41%), due to the intraoperative measurement of TM distances. The re-excisions were performed on the same day, avoiding further surgery and the patients’ discomfort. 

The re-operation rate for group B was 10.7%, while Lamb et al. reported that 15.1% of the patients underwent radicalization [17]. In a previous pilot study, the combined technique was tested on five patients, verifying oncological radicality in all the patients and the absence of migrations. Tags appeared well visible at the US scan. Patients and clinicians were asked about their satisfaction and reported excellent outcomes [7].

In the current study, the reported results suggested that the combined technique offers an important advantage for oncological radicality without significantly lengthening the surgical times. The US approach combined with the ed the safety LOCalizer^TM^ technique increased the safety and the orientation during the surgical excision and allowed mini-invasive lumpectomies. The weights and volumes of the specimens were smaller when the combined technique was performed, improving patient compliance and satisfaction with the result.

The combined localization with LOCalizer^TM^ and US is an innovative technique, and comparative studies with the golden standard are not yet available. The comparison between LOCalizer^TM^ alone and WGL showed the non-inferiority of the wireless technique.

Mc Gugin et al. performed a retrospective cohort analysis on LOCalizer^TM^ alone and WGL performances. A total of 503 procedures were included, 147 LOCalizer^TM^ (29.2%) and 356 WGL (70.8%). All intended targets were removed. Specimen volumes were similar (excisional biopsy: 8.2 vs. 8.0 cm^3^; *p* = 0.560, respectively; lumpectomy: 19.3 vs. 16.5 cm^3^; *p* = 0.494). LOCalizer^TM^ operative times were longer than WGL (57 min vs. 49 min; *p* = 0.027). Re, respectively). Re-excision rates were similar for LOCalizer^TM^ and WGL lumpectomy (19.1% vs. 16.8%; *p* = 0.615) [14].

In a study performed by DiNome et al., in Los Angeles, the LOCalizer^TM^ was utilized for 50 breast lumpectomies (33 cancers). For 93.9% of patients, the surgery was radical, and the mean volume of breast cancer was 36.3 cm^3^ [11].

Using the double localization method, in our previous study, oncological radicality was achieved in all the patients, and the mean volume of specimens was 23.4 ± 5.0 cm^3^, slightly greater than the result obtained in the current study [7]. 

An adequate comparison between the novel method and the other breast localization methods should be performed considering the oncologic radicality, the reoperation rate, the migrations, the satisfaction of clinicians and patients, and the mini-invasive approach, evaluating the specimens’ weights and volumes. Unfortunately, the reported studies studied all these parameters globally. 

Some limits were identified in the current study as well. Early studies on the LOCalizer^TM^ reported a tag placement window of only 30 days before surgery due to the increasing risk of tag migration due to changes in body habitus or breast density. More data are required on the safe duration of tag insertion and its possible use the patients undergoing neoadjuvant treatments [11,12,13]. Although the tag is magnetic resonance imaging (MRI) compatible, some artifacts will be created, and therefore it is not recommended to localize tumors evaluated by MRI prior to neoadjuvant chemotherapy. One of the main disadvantages of the LOCalizer^TM^ tag is its size. Other wireless localizing technologies are associated with smaller markers. For example, in contrast to the 5 × 1 mm steel Magseed® (Endomagnetics Inc., Cambridge, UK) pellet, the LOCalizer tag measures 11 × 2 mm in size, requiring a 2 mm skin incision prior to insertion of the applicator [18].

There are limitations to this study. This is a single-center feasibility study reporting the experience of a small cohort. Patients were carefully selected for the LOCalizer procedure.

Furthermore, larger comparative prospective studies are needed to address this issue.

## 5. Conclusions

LOCalizer^TM^ with US, in the current series, has resulted in the preferred option for the localization of non-palpable breast cancer, allowing limited resection (in weight and volume), guaranteeing excellent oncological outcomes, and great satisfaction for patients and physicians. Moreover, the combination of LOCalizer^TM^ with US does not require expensive costs because it is largely available in the surgery units.

## Figures and Tables

**Table 1:** The baseline clinical and demographic features in both Groups.

	Group A 68 Patients	Group B 28 Patients	*p*
Age (years) °	56.2 ± 8.0	58.3 ± 5.4	0.256
BMI °	28.91 ± 4.85	27.84 ± 3.31	0.249
Staging pT1a pT1b pT1c	29 (42.6%) 23 (33.8%) 16 (23.6%)	12 (42.9%) 9 (32.1%) 7 (25%)	0.759 0.873 0.878
Breast Side Right Left	37 (54.4%) 31 (45.6%)	17 (60.7%) 11 (39.3%)	0.571 0.571
Breast region Upper Lateral Quadrant Upper Medial Quadrant Lower Lateral Quadrant Lower Medial Quadrant	31 (45.6%) 15 (22.1%) 13 (19.1%) 9 (13.2%)	13 (46.4%) 5 (17.8%) 6 (21.4%) 4 (14.4%)	0.940 0.645 0.796 0.891

° Data are expressed as mean ± Standard Deviation. BMI, Body Mass Index.

**Table 2:** Tag placement in Group A and B.

	Group A 68 Patients	Group B 28 Patients	*p*
Tag placed into the NPBC	27 (39.7%)	11 (39.2%)	0.969
Tag placed near the NPBC	22 (32.4%)	5 (17.9%)	0.151
Tag placed in part into the NPBC	19 (27.9%)	12 (42.9%)	0.155

**Table 3:** Postoperative pathological outcomes and satisfaction scale for Group A and B.

	Group A 68 Patients	Group B 28 Patients	*p*
Oncologic radicality at definitive pathological exam	68 (100%)	25 (89.2%)	0.006 *
Migration rate	1 (1.5%)	0	0.518
Operative time (minutes)	15.7	14.5	0.279
Specimens volume (cm^3^)	13.2 ± 0.6	16.1 ± 1.4	0.003 *
Specimensweight (g)	21.8 ± 2.2	24.4 ± 1.8	0.004 *
Patients’ Satisfaction (Likert)	9.2	8.1	0.003 *
Clinicians’ satisfation (Likert)	9.7	7.8	0.003 *

* Statistical Significant.

## Data Availability

Not applicable.
